# Dietary ketone ester attenuates the accretion of adiposity and liver steatosis in mice fed a high-fat, high-sugar diet

**DOI:** 10.3389/fphys.2023.1165224

**Published:** 2023-04-11

**Authors:** Kelsey A. Rushing, Mickey L. Bolyard, Taylor Kelty, Nicole Wieschhaus, Gregory Pavela, R. Scott Rector, Eric P. Plaisance

**Affiliations:** ^1^ Department of Nutrition Sciences, University of Alabama at Birmingham, Birmingham, AL, United States; ^2^ Department of Human Studies, University of Alabama at Birmingham, Birmingham, AL, United States; ^3^ Research Service, Harry S. Truman Memorial Veterans’ Hospital, Department of Nutrition and Exercise Physiology, Medicine—Division of Gastroenterology and Hepatology, University of Missouri, Columbia, MO, United States; ^4^ Department of Health Behavior, University of Alabama at Birmingham, Birmingham, AL, United States

**Keywords:** ketone ester supplementation, energy balace, obesity, NAFLD, high fat high sucrose diet

## Abstract

**Objective:** The ketone diester, R,S-1,3-butanediol diacetoacetate (BD-AcAc_2_), attenuates the accretion of adiposity and reduces hepatic steatosis in high-fat diet-induced obese mice when carbohydrate energy is removed from the diet to accommodate energy from the ester. Reducing carbohydrate energy is a potential confounder due to the well-known effects of carbohydrate restriction on components of energy balance and metabolism. Therefore, the current investigation was designed to determine whether the addition of BD-AcAc_2_ to a high-fat, high-sugar diet (with no reduction in carbohydrate energy) would attenuate the accretion of adiposity and markers of hepatic steatosis and inflammation.

**Methods:** Sixteen 11-week-old male C57BL/6J mice were randomized to one of two groups for 9 weeks (n = 8 per group): 1) Control (CON, HFHS diet) or 2) Ketone ester (KE, HFHS diet + BD-AcAc_2_, 25% by kcals).

**Results:** Body weight increased by 56% in CON (27.8 ± 2.5 to 43.4 ± 3.7 g, *p* < 0.001) and by 13% in KE (28.0 ± 0.8 to 31.7 ± 3.1 g, *p* = 0.001). Non-alcoholic fatty liver disease activity scores (NAS) for hepatic steatosis, inflammation, and ballooning were lower in the KE group compared to CON (*p* < 0.001 for all). Markers of hepatic inflammation [*Tnfα* (*p* = 0.036); *Mcp1* (*p* < 0.001)], macrophage content [(Cd68 (*p* = 0.012)], and collagen deposition and hepatic stellate cell activation [(*αSma* (*p* = 0.004); *Col1A1* (*p* < 0.001)] were significantly lower in the KE group compared to CON.

**Conclusion:** These findings extend those of our previous work and show that BD-AcAc_2_ attenuates the accretion of adiposity and reduces markers of liver steatosis, inflammation, ballooning, and fibrosis in lean mice placed on a HFHS diet where carbohydrate energy was not removed to accommodate energy from addition of the diester.

## 1 Introduction

Consumption of a high-fat, high-sugar (HFHS) diet is a major contributor to the incidence of obesity and related metabolic diseases (Wright and Aronne, 2012; Gershuni et al., 2018), including non-alcoholic fatty liver disease (NAFLD) (Romero-Gomez et al., 2017). Calorie restriction (CR) is a well-established strategy to reduce body weight and adiposity. However, long-term success is often tempered by physiological responses to decreased energy intake, such as increased appetite and energy intake and decreased energy expenditure, which blunt expected weight loss and promotes weight regain (Redman and Ravussin, 2011; Most and Redman, 2020).

High-fat, low-carbohydrate ketogenic diets (KD) produce weight loss through mechanisms that likely involve increases in circulating ketones and/or decreases in circulating insulin (Kennedy et al., 2007; Hall et al., 2016; Ebbeling et al., 2018). KD-mediated reductions in body weight may be attributed to reduced appetite and energy intake (Deemer et al., 2020a; Antonio Paoli et al., 2021), maintenance of energy expenditure (Deemer et al., 2019; Hall, 2019; Antonio Paoli et al., 2021; Ludwig et al., 2021), lower concentrations of circulating insulin (Kennedy et al., 2007; Luukkonen et al., 2020; Antonio Paoli et al., 2021), and maintenance of skeletal muscle mass (Kennedy et al., 2007; Antonio Paoli et al., 2021). Studies in humans show that very low-calorie, high-fat, and normocaloric KDs may also be treatment options for NAFLD. While mechanisms have not been fully elucidated, improvements in markers of hepatic steatosis (Tendler et al., 2007; Perez-Guisado and Munoz-Serrano, 2011; European Association for the Study of the Liver et al., 2016; Mardinoglu et al., 2018; Luukkonen et al., 2020) and decreases in hepatic insulin (Tendler et al., 2007; Luukkonen et al., 2020), inflammation (Tendler et al., 2007), and fibrogenesis have been observed (Tendler et al., 2007). However, poor compliance has led to varying degrees of long-term success with KD (Kennedy et al., 2007; Hall et al., 2016; Antonio Paoli et al., 2021). The robust and consistent beneficial effects of KD on components of energy balance and metabolism have spurred interest in pursuing nutritional ketosis exogenously.

The ketone ester, R,S-1,3-butanediol diacetoacetate (BD-AcAc_2_), increases circulating ketone concentrations in rodents from 0.5 to 1.0 mM (Poff et al., 2014; Ari et al., 2016; Kesl et al., 2016; Davis et al., 2019). Studies from our laboratory show that BD-AcAc_2_ decreases body weight and adiposity in obese mice on a high-fat diet (HFD) (Davis et al., 2019) and attenuates weight gain in lean mice on a low-fat diet (LFD) (Deemer et al., 2019). A follow-up investigation demonstrated that BD-AcAc_2_ significantly reduced histological hepatic steatosis, inflammation, and NAFLD activity score (NAS) on a HFD (Moore et al., 2021). In addition, BD-AcAc_2_ decreased hepatic expression of profibrotic markers, increased markers of anti-inflammatory M2 macrophages, and reduced pro-inflammatory markers (Moore et al., 2021).

In previous studies, carbohydrate energy was removed to accommodate the energy content of BD-AcAc_2_ to create an isocaloric control diet (Davis et al., 2019; Deemer et al., 2019; Moore et al., 2021). Reducing carbohydrate energy is a potential confounder due to the well-known effects of carbohydrate restriction on components of energy balance and metabolism. Therefore, the current investigation was designed as a proof-of-concept study to determine whether the addition of BD-AcAc_2_ to a high-fat, high-sugar diet (with no reduction in carbohydrate energy) would attenuate the accretion of adiposity and markers of hepatic steatosis, fibrosis, and inflammation.

## 2 Materials and methods

### 2.1 Animals and diets

Sixteen male C57BL/6J mice were purchased from The Jackson Laboratory (Bar Harbor, ME) at 9 weeks of age and fed standard rodent chow for 1 week upon arrival to the UAB animal facilities. After 7 days of acclimation, mice were placed *ad libitum* on a HFHS diet containing 40% kcals from fat as lard, 40% carbohydrate as sucrose, and 20% protein as casein (Dyets Inc, #104925) for 1 week. Mice were then randomly assigned to one of two groups using a random number generator for an additional 9 weeks (n = 8 per group): 1) Control (CON; remain on HFHS; Dyets Inc, #104925) and 2) Ketone Ester (KE; HFHS +25% ketone ester by kcals; Dyets Inc, #104926) (See Supplement). BD-AcAc_2_ was a gift from Disruptive Enterprises (Durham, NC).

Mice were single-housed in shoebox cages with filter tops, wood-chip bedding, and shredded paper nesting for the duration of the study. Cages, bedding, feeding containers, and water were changed monthly or as needed. Animals were maintained on a standard 12:12 light-dark cycle beginning at 0600h in temperature-controlled chambers at 22°C–23°C. All procedures and conditions were approved by the UAB Institutional Animal Care and Use Committee and conformed to the care procedures employed by the UAB Animal Resources Program.

### 2.2 Body weight and food intake

Body weight was measured daily. Animals were fed powdered diets *ad libitum* daily from feeding jars (Dyets, Inc, Bethlehem, PA). The manufacturer performed bomb calorimetry to provide energy content of the diets, with energy intake calculated as 4.537 kcal/g for the CON diet and 5.671 kcal/g for the KE diet.

### 2.3 Body composition and indirect calorimetry

Body composition was measured using quantitative magnetic resonance (QMR; EchoMRI 3-in-1 version 2013; Echo Medical Systems, Houston, TX. Energy expenditure and locomotor activity were measured during the last week of the study using a TSE PhenoMaster indirect calorimetry system (TSE Instruments, Chesterfield, MO) in a temperature-controlled room at 22°C–23°C following protocols previously described (Davis et al., 2019).

### 2.4 Tissue collection and handling

Mice were fasted for two hours at 6:00 a.m. and were euthanized by decapitation in a randomized sequence two hours later. Whole blood was collected from the trunk into 1.5 mL centrifuge tubes and immediately placed on ice for at least 30 min to allow for clotting prior to centrifugation at 1,500 *g* at 4°C for 10 min. Serum was isolated and stored at −80°C until analysis. Tissues were isolated, weighed, and snap-frozen in liquid nitrogen at −80 °C until analysis.

### 2.5 Histology

An aliquot of snap-frozen liver was placed in 10% formalin, then embedded in paraffin, serially sliced, and stained with hematoxylin and eosin (H&E) to examine liver morphology (histological steatosis, inflammation, and hepatocyte ballooning) by IDEXX RADIL (Columbia, MO). Images were obtained using an Olympus BX43 microscope (Waltham, MA) at ×10 magnification. Liver specimens were graded and scored for differences in NAS (comprising fat accumulation, inflammation, and hepatocellular ballooning) by an individual blinded to the study groups for differences according to the Brunt Scale (Brunt et al., 1999).

### 2.6 Biochemical assays

Serum glucose and non-esterified fatty acid concentrations were determined using colorimetric assays (Cayman Chemical, Ann Arbor, MI, and Sigma, St. Louis, MO, respectively). R-βHB concentration was measured from whole blood using a hand-held device (Keto-Mojo, Napa, CA). Serum insulin was measured using an ELISA kit from Alpco (Salem, NH). Quantitative insulin-sensitivity check index (QUICKI) and homeostasis model assessment for insulin resistance (HOMA-IR) were calculated as previously described (Matthews et al., 1985; Katz et al., 2000).

### 2.7 Western blot analysis

Whole tissue liver homogenates were used for Western blot analysis. Cell lysates were initially prepped in Triton X-100. Laemmli gel loading buffer was added to the lysate (1 μg/uL) and boiled at 100°C for 10 min. Membranes were incubated for one hour in blocking solution [5% dry milk in Tris-buffered saline (TBS), 0.1% Tween 20 buffer] after gel electrophoresis and transfer. Membranes were incubated overnight in the primary antibody of interest. Membranes were then washed three times for five minutes each with TBS-Tween 20 buffer and placed in a HRP-conjugated secondary antibody that was either anti-rabbit or anti-mouse specific (Nos. 7074 and 7076; Cell Signaling Technology, Danvers, MA). Blots (n = 8/group) were quantified using densiometric analysis (Image Lab 4.0). Differences in protein loading were controlled with amido-black staining. Corrections for differences in protein loading and transfer of all band intensities were quantified by laser densitometry using the total protein staining from each lane.

### 2.8 mRNA expression analysis

A commercially available kit (RNEasy; No. 74104, Germantown, MD) was used to extract RNA from liver and to synthesize a cDNA library (Sigma, St. Louis, MO). A nanodrop spectrometer was used to assess the purity and quality of RNA and cDNA samples. qPCR using SYBR Green (No. 172–5121, Bio-Rad Laboratories) and primer pairs (BRAND) were performed. Data are presented relative to *GAPDH* using the 2^−ΔΔCT^ method. See Supplement for primer sequences.

### 2.9 Statistical analysis

Kolmogorov-Smirnov and Shapiro-Wilk tests were conducted on all data before analysis to examine distribution of the data. Independent samples t-tests were used to determine differences in tissue weights, NAS, mRNA, and protein. Mann-Whitney non-parametric tests were used for data that were non-normally distributed, which includes, R-βHB, locomotor activity (total, light, and dark), insulin sensitivity (insulin concentrations and HOMA-IR), mRNA expression of *Arg1, Il-1β, Col1A1, Mcp1, G6Pase,* and *GAPDH*, steatosis, ballooning, inflammation, and protein content of PDGFB, TRAIL, αSMA, and CD11b. Mean weekly body weight and energy intake were analyzed using a 2 (group) x 10 (time) repeated-measures ANOVA. The Huynh-Feldt correction was used to report these interactions’ significance due to violation of Mauchly’s test of sphericity. Differences in lean body mass (LBM) and fat mass (FM) were analyzed by 2 (group) x 2 (time) repeated measures ANOVA. Group differences in REE and TEE (kcal/mouse/day) were determined by ANCOVA, with LBM and FM included as covariates as previously described (Tschop et al., 2011; Davis et al., 2019; Deemer et al., 2019; Deemer et al., 2020b). All analyses were conducted with an n of 8 per group except for energy intake. During the first 2 weeks of the experimental phase of the study, food intake for two mice in the control group was eliminated due to a behavior pattern in which the mice removed all food from the feeding jar, which made it impossible to measure energy intake accurately. Significance was set *a priori* at *p* < 0.05, and data are expressed as means ± SD unless otherwise noted. All statistical analyses were conducted using IBM SPSS Statistics (version 29.0; IBM Corp., Armonk, New York).

## 3 Results

### 3.1 Dietary BD-AcAc_2_ attenuates increases in body weight and adiposity in the presence of a HFHS diet

**FIGURE 1:**
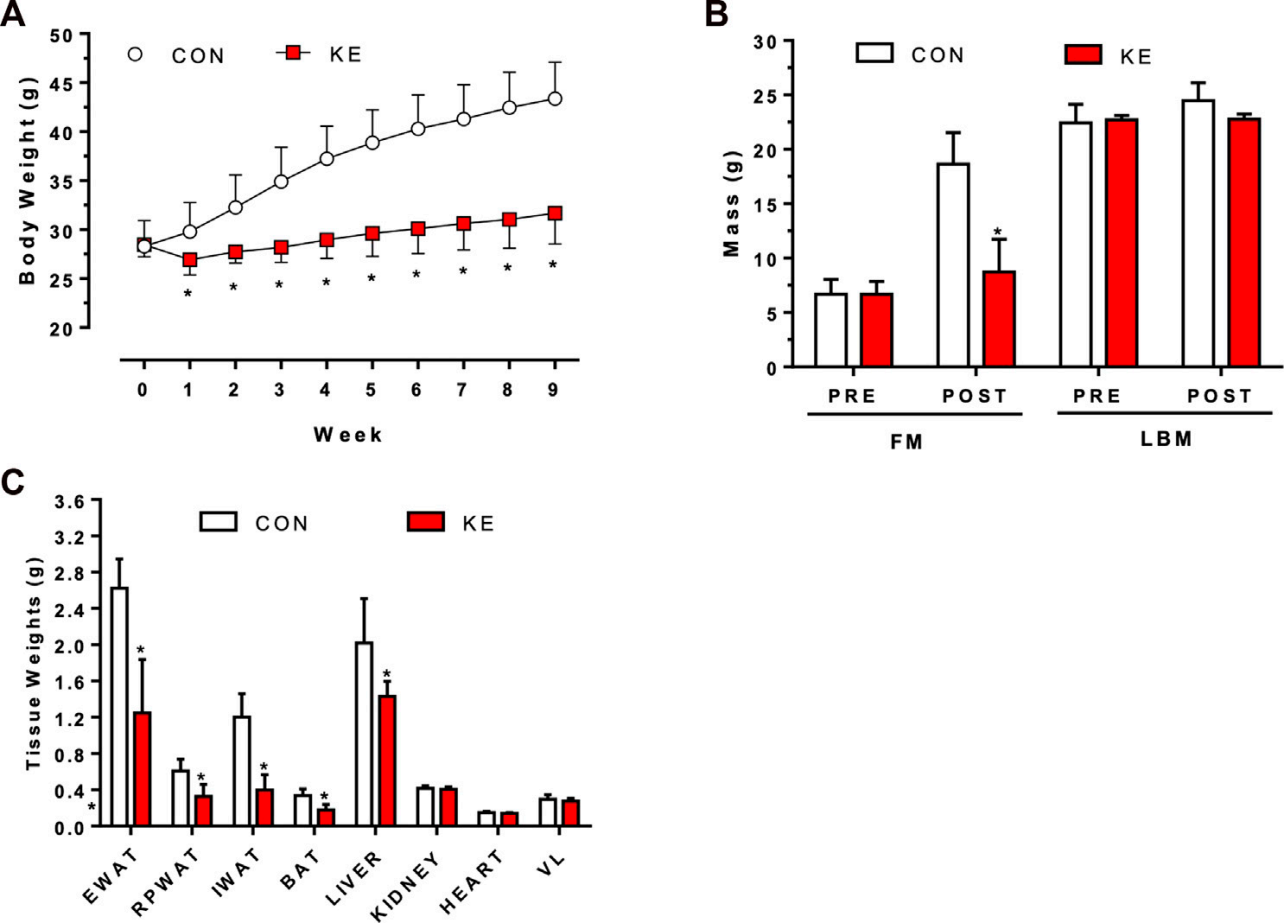
Differences in body weight, body composition, and tissue weights after 9 weeks of Control (CON; HFHS) or Ketone Ester (KE; HFHS +25% ketone ester by kcals) diets. **(A**) Body weight responses across 9 weeks *p < 0.05, CON vs KE beginning at week 1.**(B)** Pre- and Post-Fat Mass (FM) and Lean Body Mass (LBM) measurements. *p < 0.001. FM gain in the CON group was significantly greater than the KE group. *p < 0.001.**(C)** Final tissue weights for epididymal (EWAT), retroperitoneal (RPWAT), and inguinal (IWAT) white adipose tissue, brown adipose tissue (BAT), liver, kidney, heart, and vastus lateralis (VL). *p < 0.05 for all adipose tissue weights and liver weight. Values are presented as means ± SD.

There was a significant interaction between group and time on body weight (Figure 1A; *p* < 0.001). Specifically, during weeks 1 through 9, mean body weight was significantly lower in the KE group compared to CON (*p* < 0.05). Between group differences in body weight were primarily attributed to a greater accretion of total adiposity in CON compared to KE (Figure 1B; *p* < 0.001). Epididymal, retroperitoneal, and inguinal white adipose tissue weights confirm increased adiposity in the CON group, as the KE group accumulated significantly less visceral and subcutaneous fat (Figure 1C; *p* < 0.05 for all). LBM increased in the CON group (*p* < 0.001) but was unchanged from baseline in the KE group (*p* > 0.05).

### 3.2 Effects of BD-AcAc_2_ on energy intake and energy expenditure

**FIGURE 2:**
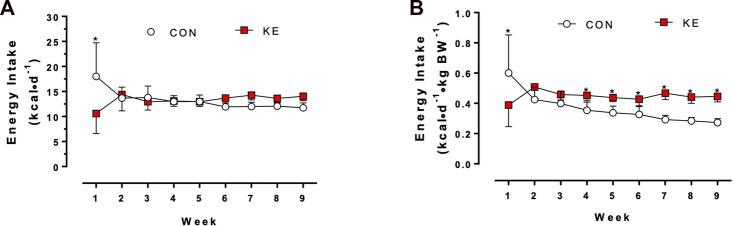
Differences in energy intake after 9 weeks of Control (CON; HFHS) or Ketone Ester (KE; HFHS +25% ketone ester by kcals) diets. **(A)** Energy intake in kilocalories per mouse per day (kcal/mouse/d). *p < 0.001 at week 1; *p* > 0.05 for the remainder of the experimental phase. **(B)** Energy intake in kilocalories per gram body weight (kcal/g BW). *p < 0.05 beginning at week 4 and for the remainder of the experimental phase. Values are presented as means ± SD.

There was a significant interaction between group and time on energy intake (Figure 2A; *p* = 0.007). Mean energy intake was significantly lower in the KE group compared to CON (*p* = 0.046) 1 week after initiation of the diet. There were no significant differences in mean energy intake between groups in weeks 2 through 5. Beginning in week 6, mean energy intake was significantly higher in KE compared to CON (*p* < 0.05).

We also examined body weight-adjusted energy intake. There was a significant interaction between group and time on relative energy intake (Figure 2B; *p* = 0.010). Specifically, beginning in week 4, mean relative energy intake was significantly higher in KE compared to CON (*p* < 0.05).

TEE and REE were significantly lower in KE compared to CON (Table 1; *p* < 0.001). However, there were no group differences in mean 24-h TEE (*p* = 0.610) or REE (*p* = 0.200) after adjustment for FM and LBM. An analysis was conducted to further explore the data during the 12-hour dark cycle, when mice consume most of their energy, and during the 12-hour light cycle. Adjusted TEE and REE were not statistically different between groups for the dark or light cycle but were directionally higher in the KE-fed mice during the dark cycle and lower during the light cycle.

**TABLE 1:** Resting and total energy expenditure.

	Daily (kcal)		Dark cycle (kcal/h)		Light cycle (kcal/h)	
	REE	TEE	REE	TEE	REE	TEE
CON						
Unadjusted	9.8 ± 0.9^a^	12.8 ± 1.0^a^	0.44 ± 0.04^a^	0.56 ± 0.04^a^	0.42 ± 0.04^a^	0.50 ± 0.04^a^
^a^Adjusted	9.5 ± 0.4^a^	12.2 ± 0.3^a^	0.42 ± 0.02^a^	0.53 ± 0.01^a^	0.40 ± 0.02^a^	0.48 ± 0.02^a^
KE						
Unadjusted	8.3 ± 0.5^b^	11.2 ± 0.5^b^	0.41 ± 0.03^b^	0.51 ± 0.03^b^	0.35 ± 0.02^b^	0.42 ± 0.02^b^
^a^Adjusted	8.6 ± 0.4^a^	11.9 ± 0.3^a^	0.43 ± 0.02^a^	0.55 ± 0.01^a^	0.36 ± 0.02^a^	0.43 ± 0.02^a^

^a^ Values were adjusted for Fat Mass (FM) and Lean Body Mass (LBM) using ANCOVA., Values are represented as means ± SD, from 24-h or 12-h (dark and light cycle) continuous indirect calorimetry performed following the 9-week intervention. Mice were acclimated in the TSE, system for 48 h before gases were measured for 24 h. Values with different letters are significantly different (*p* < 0.05).

**FIGURE 3:**
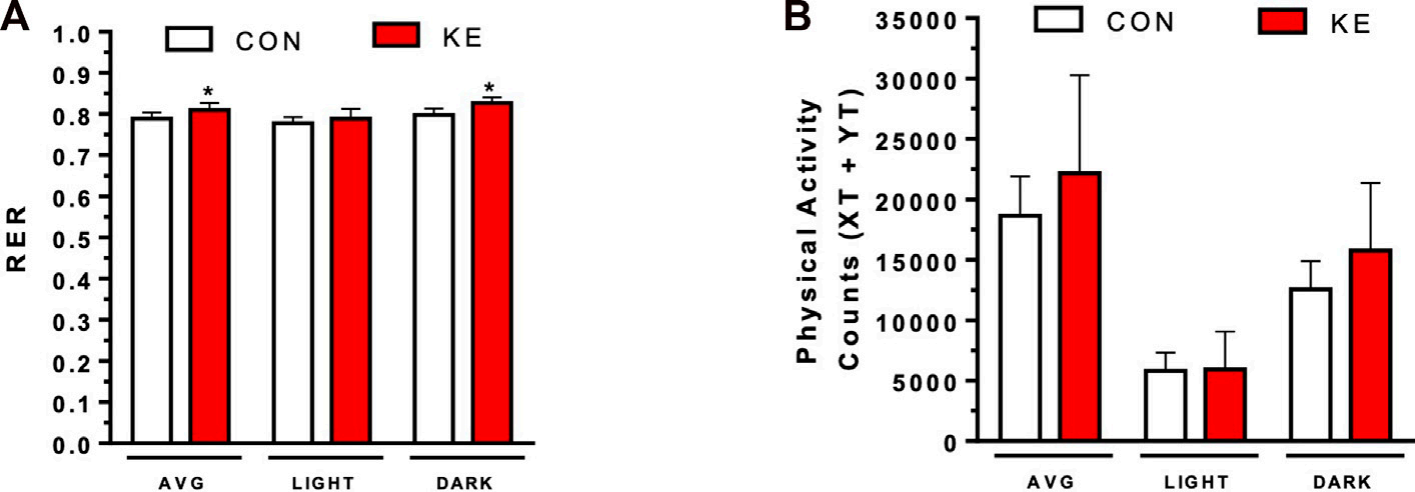
Final respiratory exchange ratio (RER) and physical activity counts measurements. **(A)** RER was calculated as VCO_2_/VO_2_. P < 0.05, total and dark RER. **(B)** Physical activity counts were measured using XT and YT activity. Values are presented as means ± SD. *p* < 0.05, * indicates significant differences between groups.

The KE group had a significantly higher 24-h mean respiratory exchange ratio (RER) (*p* = 0.024) and dark cycle RER (*p* = 0.002) compared to CON (Figure 3A). In contrast, RER during the light cycle was not significantly different between groups (*p* = 0.314). There were also no significant differences between groups in total, dark, or light locomotor activity (Figure 3B; *p* > 0.05).

### 3.3 BD-AcAc_2_ attenuates hepatic steatosis, NAS, and markers of hepatic stellate cell activation and fibrosis

**FIGURE 4:**
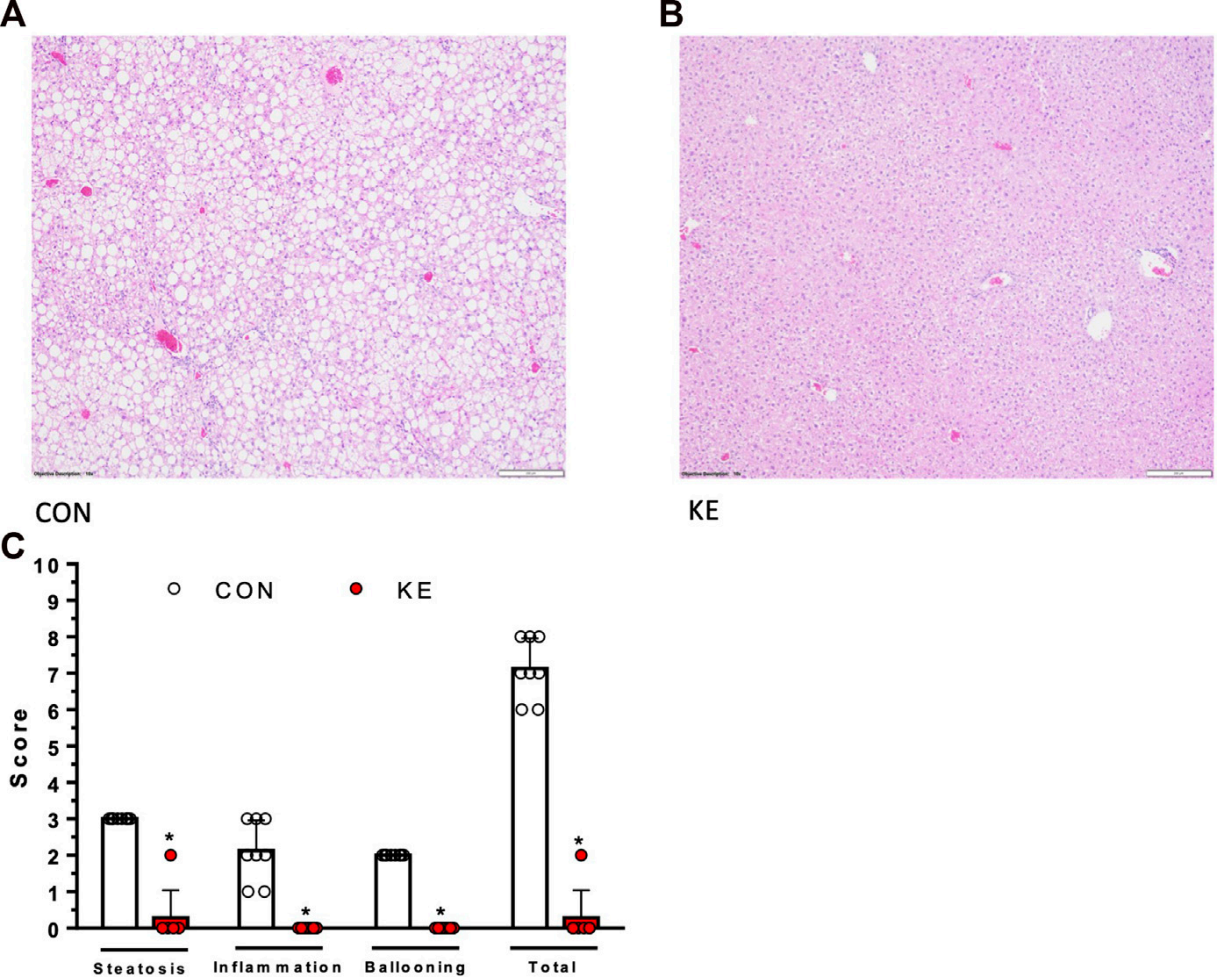
Differences in liver phenotype assessed at study completion. **(A), (B)** Representative hematoxylin and eosin images of liver morphology. CON group has greater accumulation of lipids compared to KE. **(C)** Differences in non-alcoholic fatty liver disease activity score (NAS; fat accumulation, inflammation, and hepatocellular ballooning), calculated by grading and scoring liver specimens. Values are presented as means ± SD. *p* < 0.05, * indicates significant differences between groups.

**FIGURE 5:**
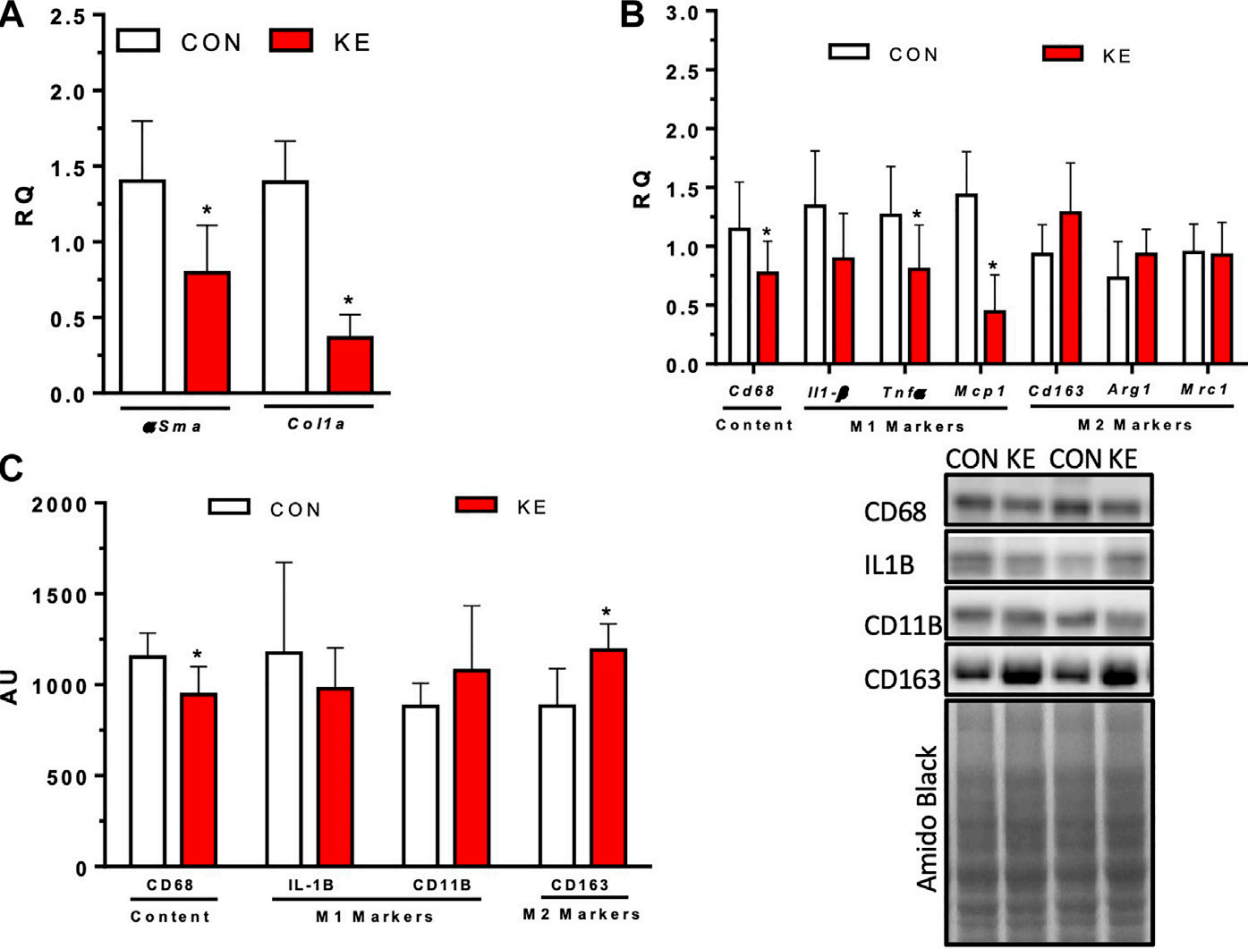
Markers of hepatic stellate cell activation, fibrosis, and hepatic inflammation after 9 weeks of CON or KE diets. **(A)** Hepatic markers of fibrosis, *αSma* and *Col1A1* mRNA expression. **(B)** mRNA expression of inflammatory hepatic marker *Cd68*, pro-inflammatory hepatic M1 markers *Il-1β, Tnfα,* and *Mcp1*, and anti-inflammatory hepatic M2 markers *Cd163, Arg1*, and *Mrc1*. **(C)** Protein content of inflammatory hepatic marker CD68, pro-inflammatory hepatic M1 markers Il-1β and CD11b, and anti-inflammatory hepatic M2 marker Cd163. Values are presented as means ± SD. *p* < 0.05, * indicates significant differences between groups.

The KE group had significantly lower hepatic steatosis, inflammation, ballooning, and total NAS compared to CON (Figures 4A–C, *p* < 0.001 for all). *αSma* (*p* = 0.004) and *Col1A1* (*p* < 0.001) mRNA expression, markers of collagen deposition and hepatic stellate cell (HSC) activation, were significantly lower in KE compared to CON (Figure 5A). However, hepatic protein expression of the fibrogenesis markers, PDGFB, MMP9, and MMP2, were not significantly different between groups (*p* > 0.05 for all, data not shown).

### 3.4 Effects of BD-AcAc_2_ supplementation on markers of hepatic inflammation

*Cd68* mRNA expression (*p* = 0.047) and CD68 protein content (*p* = 0.012) were significantly lower in the KE group compared to CON (Figures 5B, C). Hepatic pro-inflammatory M1 macrophage marker *Tnfα* (*p* = 0.036) and *Mcp1* (*p* = 0.000) mRNA expression were also lower in the KE group compared to CON. There were no significant differences between groups in hepatic pro-inflammatory M1 Il-1β mRNA expression or IL-1β, TRAIL, or CD11b protein content (*p* > 0.05). mRNA expression of *Cd163* was higher in the KE group compared to CON but did not achieve statistical significance (Figure 5B; *p* = 0.062). However, CD163 protein expression was significantly higher in the KE group compared to CON (Figure 5C; *p* = 0.004). No statistically significant differences were observed between groups for other M2 markers, *Arg1* and *Mrc*1 (Figure 5B; *p* > 0.05).

### 3.5 Exploratory analysis of DNL, mitochondrial biogenic markers, and glucose metabolism

**FIGURE 6:**
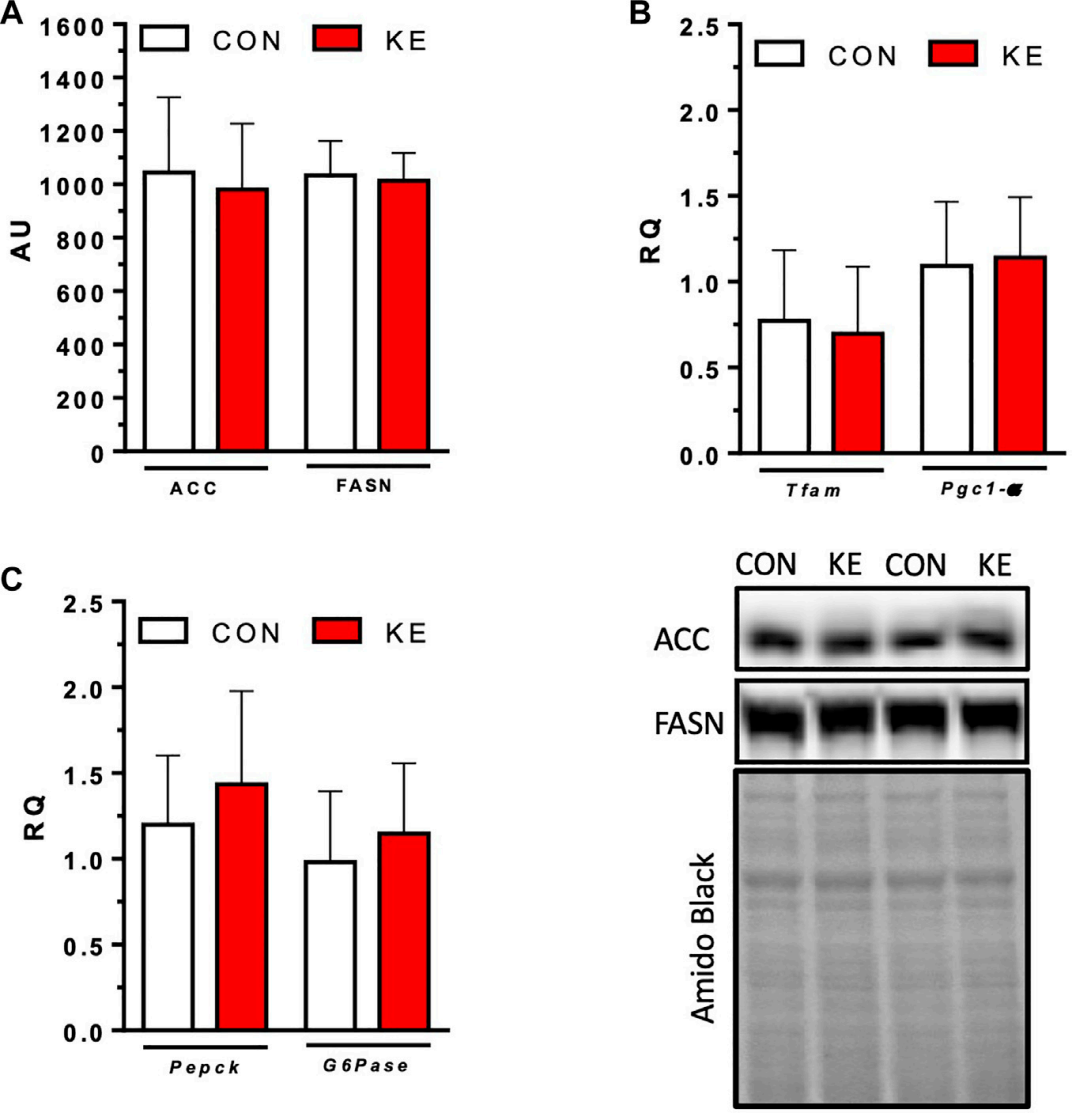
Markers of *de novo* lipogenesis (DNL) and mitochondrial biogenesis. **(A)** Protein content of hepatic ACC and FASN. **(B)** mRNA expression of key components of DNL, *Pgc1-α* and *Tfam*. **(C)** mRNA expression of markers of gluconeogenesis*, Pepck* and *G6pase*. Values are presented as means ± SD. *p* < 0.05, * indicates significant differences between groups.

The present investigation was a proof-of-concept study designed to test our hypothesis that BD-AcAc_2_ would attenuate hepatic lipid accumulation and inflammation on a HFHS diet. The phenotype observed here and in our previous studies (Davis et al., 2019; Deemer et al., 2019; Moore et al., 2021) highlights the need to examine potential molecular mechanisms. Thus, we examined mRNA expression of key components of *de novo* lipogenesis (DNL) to explore whether lower liver lipid content in the KE group was due to decreased DNL expression. Hepatic protein content of ACC and FASN were not significantly different between groups (Figure 6A; *p* > 0.05 for both). mRNA expression of *Pgc1-α* (*p* = 0.796) and *Tfam* (*p* = 0.716) (Figure 6B) were also not significantly different between groups, suggesting that hepatic markers of mitochondrial biogenesis were unaltered by BD-AcAc_2_ administration.

R-βHB concentrations were higher in the KE group compared to CON (Table 2). S-βHB and AcAc concentrations that require more sophisticated experimental approaches were not measured and likely contribute to a significant underestimation of total circulating ketones. Serum glucose and insulin concentrations were measured to examine effects of the ketone ester on glucose metabolism. Glucose (*p* = 0.371) did not reach statistical significance for differences between groups. However, circulating concentrations of insulin were significantly lower in KE compared to CON (Table 2) (*p* = 0.038). QUICKI scores were significantly higher in the KE group than in the CON (*p* = 0.026), while HOMA-IR was significantly lower (*p* = 0.021). mRNA expression of *Pepck* (*p* = 0.343) and *G6pase* (*p* = 0.381) were not significantly different between groups (Figure 6C), providing initial evidence that a reduction in hepatic gluconeogenesis may not contribute to the improvement in markers of insulin sensitivity.

**TABLE 2:** Blood markers.

	CON	KE
Insulin (ng·mL^-1^)	4.3 ± 2.8	2.3 ± 1.1^a^
Glucose (mg·dL^-1^)	215 ± 35	202 ± 22
NEFA (nmol·µL^-1^)	31.5 ± 6.6	38.4 ± 7.8
R-βHB (mM)	0.1 ± 0.0	0.338 ± 0.3

^a^Values are means ± SD. * = significance set at *p* < 0.05.

NEFA, Non-esterified fatty acids.

## 4 Discussion

BD-AcAc_2_ reduces (Ciarlone et al., 2016; Davis et al., 2019) or lowers the accretion of (Poff et al., 2014; Kesl et al., 2016; Deemer et al., 2019; Deemer et al., 2020b) body weight and adiposity. A more recent publication from our group showed that BD-AcAc_2_ decreased hepatic steatosis, fibrosis, and inflammatory markers compared to *ad libitum* HFD-fed obese controls (Moore et al., 2021). In each of these studies, 25%–30% of carbohydrate energy was removed to produce isocaloric diets (Davis et al., 2019; Deemer et al., 2019; Deemer et al., 2020b; Moore et al., 2021), the metabolic effects of which cloud interpretation of the results. The purpose of this investigation was to examine whether adding BD-AcAc_2_ to the diet without removing carbohydrate energy and in the presence of a HFHS diet would attenuate the accretion of adiposity and markers of NAFLD. Our findings indicate that ketone-fed mice on a HFHS diet accrued less adiposity than controls and had lower NAS (inflammation, steatosis, and ballooning), fibrosis, and HSC activation.

HFHS diets produce positive energy balance leading to obesity and obesity-related metabolic diseases such as NAFLD. In the current investigation, mice in the CON group developed a phenotype consistent with NAFLD. However, mice in the KE group gained only a fraction of the body weight and adiposity observed in the CON group, indicating that BD-AcAc_2_ produced an effect on one or more components of energy balance. Absolute energy intake was similar between groups despite significant differences in body weight and adiposity. While reporting energy intake relative to body weight may not be appropriate due to differences in energy requirements of various tissues (Kaiyala and Schwartz, 2011), a 22% higher relative energy intake suggests that energy needs were higher in the KE group to maintain body composition. Previous studies show that energy intake is either decreased (Davis et al., 2019; Deemer et al., 2019) or unchanged (Deemer et al., 2019; Deemer et al., 2020b). Davis and colleagues (Davis et al., 2019) reported that BD-AcAc_2_-fed obese mice on a HFD experienced a 26% decrease in energy intake. However, mice in the KE group had significantly lower body weight and adiposity than a pair-fed group despite receiving similar energy provisions. In contrast, lean mice placed on a LFD with BD-AcAc_2_ administration had similar absolute and body-weight relative energy intake compared to controls (Deemer et al., 2019). These findings and those from the current investigation indicate that a decrease in energy intake and, for the first time, removal of carbohydrate energy (in the presence of a HFHS diet) is unnecessary to limit the accretion of adiposity. While the mechanisms responsible for the robust differences in body weight and adiposity observed with mice receiving the ketone diester remain elusive, we predict that energy expenditure is likely higher during the transition to ketone diester administration. By measuring energy expenditure at the completion of the study in each of our previous investigations, including this one, we are likely missing early changes in components of energy intake, energy expenditure, and energy loss. Upcoming studies are planned to thoroughly interrogate early responses that are likely contributing to the body composition phenotype.

Adjusted 24-hour TEE and REE were similar between groups when measured at completion of the study. While dark cycle TEE and REE were not statistically different between groups, there was a noticeable pattern of higher TEE and REE when mice consume over 70% of their daily energy. In contrast, mice in the KE group had statistically similar but numerically lower TEE and REE during the light cycle compared to CON. BD-AcAc_2_-fed obese mice on a HFD had significantly higher TEE and REE compared to pair-fed animals receiving similar energy provisions suggesting that increased energy expenditure contributed to development of the phenotype (Davis et al., 2019). As part of a LFD, BD-AcAc_2_ produced no change or even a slight decrease in energy expenditure (Deemer et al., 2019; Deemer et al., 2020b). In our previous investigation (Davis et al., 2019), we observed a transcriptional signature in brown adipose tissue (BAT), but not WAT, that was consistent with a thermogenic phenotype. Although it was not within the scope of work in this investigation, future studies are needed to explore the possibility that increasing circulating ketones with exogenous ketone esters could increase thermogenic capacity in BAT.

Ketone energy losses through breath, urine, and feces are challenging to measure in rodents and the current nutritional model, where ketones are incorporated into the diet and consumed at each feeding rather than at designated times. A previous investigation measured feces output while on a LFD and found that ketone-fed mice had 50% greater fecal loss (Deemer et al., 2020b). However, fecal energy loss explained only 25% of the difference in adiposity between groups (Deemer et al., 2020b). Although we did not collect feces in the current investigation, it will be important to establish the contribution of energy loss in future studies to define the mechanisms responsible for the body weight phenotype produced. It will also be important in future studies to determine differences in the energy content of the diester using bomb calorimetry *versus* extractable energy from the racemic mixture of butanediol.

As described above, a limitation of the current investigation is that we measured energy expenditure only during the final week of the study. The significantly higher body weight and adiposity of the CON group compared to KE require statistical adjustment using FM and LBM for comparison purposes. Future studies should focus on changes in energy expenditure during the introduction of the diet before appreciable differences emerge in body mass to determine the role of BD-AcAc_2_ on energy balance. Conducting these studies will help to ascertain the specific mechanisms related to energy expenditure or energy loss that contribute to the defense of adiposity and body weight with BD-AcAc_2_ administration in multiple conditions, including the present HFHS dietary background. It will also be important to devise methods to measure energy losses through breath and urine, as these likely contribute to the phenotype and may affect indirect calorimetry results (Battezzati and Vigano, 2001). Finally, our studies to date have only included male mice. Considering that female mice may respond differently to the diet regarding the accretion of adiposity and hepatic lipid accumulation than male mice, future studies are planned by our group to investigate sexual dimorphism using this nutritional model.

We previously reported that BD-AcAc_2_ improved markers of NAFLD in HFD-fed obese mice compared to both control and pair-fed mice (Moore et al., 2021). In the present investigation, BD-AcAc_2_ abolished the development of hepatic steatosis, inflammation, and ballooning in lean mice compared to controls on an identical HFHS diet. These results provide evidence that BD-AcAc_2_ decreases markers of NAFLD in obese mice on a HFD and lean mice placed on a HFHS diet despite similar energy intake and expenditure between groups. These findings are potentially important as they suggest mechanisms that are independent of CR or reductions in carbohydrate energy are responsible for the improved hepatic outcomes.

Another hallmark of the progression of NAFLD to NASH is hepatic inflammation, specifically, an imbalance in pro- and anti-inflammatory macrophages (Alisi et al., 2017). Ketone administration reduces inflammation in multiple tissues, such as the heart, kidney, and liver (Clarke et al., 2012; Soto-Mota et al., 2020; Soni et al., 2022). Indeed, we demonstrated that BD-AcAc_2_ lowered expression of multiple pro-inflammatory M1 markers and higher expression of M2 anti-inflammatory markers. Mitigation of hepatic inflammation in ketone-fed mice may be due to the anti-inflammatory properties of *β*-hydroxybutyrate (βHB). The anti-inflammatory effects of βHB appear to be at least in part mediated by inhibition of the NLR family pyrin domain containing 3 (NLRP3) inflammasome (Soni et al., 2022). In a mouse model of LPS-induced sepsis, mice fed a ketone monoester had reduced markers of NLRP3 activation and markers of hepatic inflammation, including *Il-1β* and *Tnfα* (Soni et al., 2022). Others (Youm et al., 2015) show that both R- and S-enantiomers of βHB (but not AcAc) inhibit the NLRP3 inflammasome by preventing K^+^ efflux and reducing ACS oligomerization and speck formation in a GPR109A (HCAR2) and histone deacetylase (HDAC) independent fashion in macrophage cell culture. In the same investigation, βHB also attenuated caspase-1 activation and IL-1β secretion in mouse models of NLRP3-mediated disease. Others (Puchalska et al., 2019) have shown that exogenous AcAc, but not βHB, ameliorated diet-induced hepatic fibrosis through mechanisms that appear linked to the oxidation of AcAc in macrophages, as evidenced by the observation that succinyl Co-transferase (SCOT) null mice (which are unable to oxidize AcAc) had increased fibrosis. These findings suggest that AcAc or βHB-mediated inhibition of inflammatory pathways may play a role in the anti-inflammatory effects of BD-AcAc_2_.

Diets high in sucrose often upregulate the expression and activity of proteins involved in DNL (Soni et al., 2022). Therefore, we measured protein expression of ACC and FASN, which are well-known markers of DNL activity. In the current investigation, BD-AcAc_2_ did not affect hepatic protein content of either ACC or FASN. More sophisticated labeling approaches using heavy water and mass spectrometry are needed, as transcriptional levels of key enzymes in the DNL cascade do not always translate into changes in enzyme activity. The HFHS diet used in this investigation would be expected to reduce endogenous ketogenesis by reducing the rate-limiting enzyme in ketogenesis, HMGCS2 (Cotter et al., 2014). Reductions in HMGCS2 expression have been shown to increase DNL in mature mice on a HFD by eliminating the capacity to siphon carbons from the TCA cycle toward ketogenesis. It is possible that exogenous ketones re-activate this pathway through mechanisms that could involve activation of HDACs. We also did not find that markers of mitochondrial biogenesis were increased in the liver. However, future studies should examine mitochondrial respiration in BAT, skeletal muscle, and liver to determine if the ketone ester alters coupled and/or uncoupled respiration.

The observations in the liver may also be attributed to the BD-AcAc_2_’s ability to attenuate the accretion of adiposity on a HFHS diet, thereby limiting ectopic deposition or production of liver fat. A close examination of the data in the current study provides evidence that an attenuation in body weight and adiposity is not required to mitigate the increase in hepatic lipid content observed in control animals. For example, one mouse in the KE group gained a similar amount of body weight and adiposity to the relatively uniform increases observed in CON mice. However, the hepatic NAS score was lower (and similar to other KE-treated mice) than the control mice. Future studies will be needed to determine whether BC-AcAc_2_ directly affects hepatic lipid metabolism and inflammation.

While circulating R-βHB concentrations were significantly higher in the KE group compared to the CON group, the measured ketone concentrations may not reflect the true level of ketosis reached throughout the experiment with the ketone ester. First, a carefully conducted fasting and re-feeding protocol was not followed, which would be necessary to control the timing of the last feeding bout more tightly. In addition, current hand-held technology does not detect S-βHB and AcAc concentrations from whole blood, and more-sophisticated approaches using expensive tandem mass spectrometry procedures were outside the scope of the current investigation. This was also not a primary outcome measure for the study, as it has been demonstrated that the ketone ester increases βHB and AcAc concentrations in rodents (Poff et al., 2014; Ari et al., 2016; Ciarlone et al., 2016; Kesl et al., 2016; Davis et al., 2019; Deemer et al., 2020b).

Circulating insulin concentrations were significantly lower, and QUICKI and HOMA-IR calculations indicate greater insulin sensitivity in the KE-fed mice with no significant differences in glucose concentrations between groups. Other approaches, such as hyperinsulinemic-euglycemic clamp with tracers, will be needed to examine whether the ketone ester improves insulin sensitivity and in what tissue(s). Although our initial findings suggest that BD-AcAc_2_ administration does not alter markers of gluconeogenesis, it will be important for future studies using tracers to fully examine hepatic glucose production.

The findings of this study, while challenging to explain mechanistically, have significant potential from a clinical and practical perspective. CR-mediated weight loss is the most used strategy to reduce NAFLD in humans and rodents. The downside is that CR is often ineffective long-term, with weight regain and metabolic dysfunction returning within the first 2–5 years (Heymsfield and Wadden, 2017). By adapting the results from our current and previous (Moore et al., 2021) investigations to humans, we could avoid the strict requirements of CR and KD. Another fascinating observation that will require additional work is that most of the weight gain in the KE-fed mice was due to a non-statistically significant increase in LBM. A recent publication from our laboratory showed that in older mice, several markers of skeletal muscle hypertrophy and atrophy are altered by BD-AcAc_2_ in a pattern suggestive of improvements in skeletal muscle quantity and quality (Roberts et al., 2022).

## 5 Conclusion

The dietary ketone ester, BD-AcAc_2_, attenuates weight and body fat gain in C57BL6 mice on a HFHS diet. Previous investigations examined the effects of exogenous ketones with dietary carbohydrates removed to produce isocaloric control diets. In the present study, adding the ketone ester to the diet without removing carbohydrate energy (in the presence of high fat and high sugar content) decreased body weight and adiposity. Furthermore, BD-AcAc_2_ completely abolished liver steatosis and markers of fibrosis and inflammation. Since there are few effective treatments for NAFLD, these findings highlight the exciting possibility for ketones in this space if observed in humans.

## Data Availability

The original contributions presented in the study are included in the article/Supplementary Materials, further inquiries can be directed to the corresponding author.
