# Supplementary material for: Dietary ketone ester attenuates the accretion of adiposity and liver steatosis in mice fed a high-fat, high-sugar diet
Source: Front Physiol. 2023 Apr 11;14:1165224. doi: 10.3389/fphys.2023.1165224 (PMC10128912; doi:10.3389/fphys.2023.1165224)
Supplement: Supplementary file 1 [file Table1.DOCX]

Supplementary Table 1. High-fat high-sugar control (CON) and ketone ester (KE) diets

| Ingredient | kcal/g | CON (g/kg) | KE (g/kg) | CON (kcal/kg) | KE (kcal/kg) |
| --- | --- | --- | --- | --- | --- |
| Casein | 3.6 | 250.0 | 250.0 | 895.0 | 895.0 |
| L-Cystine | 4.0 | 3.0 | 3.0 | 12.0 | 12.0 |
| Sucrose | 4.0 | 440.0 | 440.0 | 1760.0 | 1760.0 |
| Cornstarch | 3.6 | 0.0 | 0.0 | 0.0 | 0.0 |
| Dyetrose | 3.8 | 0.0 | 0.0 | 0.0 | 0.0 |
| Lard | 9.0 | 200.0 | 200.0 | 1800.0 | 1800.0 |
| Cellulose | 0.0 | 49.5 | 49.5 | 0.0 | 0.0 |
| Mineral Mix #210025 | 0.9 | 35.0 | 35.0 | 30.8 | 30.8 |
| Vitamin Mix #310025 | 3.9 | 10.0 | 20.0 | 38.7 | 38.7 |
| Choline Bitartrate | 0.0 | 2.5 | 2.5 | 0.0 | 0.0 |
| Sodium Saccharin | 0.0 | 10.0 | 10.0 | 0.0 | 0.0 |
| Ketone Ester | 5.8 | 0.0 | 195.6 | 0.0 | 1134.3 |
| Total |  | 1000.0 | 1195.6 | 4537.0 | 5671.0 |
