# Supplementary material for: Dietary ketone ester attenuates the accretion of adiposity and liver steatosis in mice fed a high-fat, high-sugar diet
Source: Front Physiol. 2023 Apr 11;14:1165224. doi: 10.3389/fphys.2023.1165224 (PMC10128912; doi:10.3389/fphys.2023.1165224)
Supplement: Supplementary file 2 [file Table2.DOCX]

Supplementary Table 2. Primer Sequences

| *Gene Name* | *Forward (5’-3’)* | *Reverse (3’-5’)* |
| --- | --- | --- |
| CD68 | CAATTCAGGGTGGAAGAAAG | TCTGATGTAGGTCCTGTTTG |
| CD163 | AGTCTGCTCACGATACATAG | TCCTTCTGGAATAGATTGGG |
| GAPDH | CTTCAACAGCAACTCCCACTC | GCCGTATTCATTGTCATACCAGG |
| G6Pase | AGGAAGGATGGAGGAAGGAA | TGGAACCAGATGGGAAAGAG |
| MCP-1 | AGCTGTAGTTTTTGTCACCAAGC | GTGCTGAAGACCTTAGGGCA |
| PEPCK | CCACAGCTGCTGCAGAACAC | GAAGGGTCGCATGGCAAA |
| TNFα | GTGACAAGCCTGTAGCCCAC | GCAGCCTTGTCCCTTGAAGA |
